# Treatment Pattern of Antithrombotic Therapy over Time after Percutaneous Coronary Intervention in Patients with Atrial Fibrillation in Real-World Practice in Korea

**DOI:** 10.3390/healthcare9091185

**Published:** 2021-09-09

**Authors:** Seongwook Han, Sola Han, Sung-Won Jang, Myung-Yong Lee, Young-Keun On, Oh Young Bang, Ji-Min Lee, Yoo-Jung Park, Ji-Soo Shin, Seongsik Kang, Hae Sun Suh, Young-Hoon Kim

**Affiliations:** 1 Division of Cardiology, Department of Internal Medicine, Dongsan Hospital, Keimyung University School of Medicine, Daegu 42601, Korea; swhan@dsmc.or.kr; 2 Pharmaceutical Economics, Big Data Analysis & Policy, College of Pharmacy, Kyung Hee University, Seoul 02447, Korea; sola.han@khu.ac.kr; 3 Division of Cardiology, Department of Internal Medicine, Catholic University of Korea, Seoul 06591, Korea; clement@naver.com; 4 Division of Cardiology, Department of Internal Medicine, Dankook University, Cheonan 31116, Korea; mel_lee@dankook.ac.kr; 5 Department of Cardiology, Samsung Medical Center, Sungkyunkwan University School of Medicine, Seoul 06355, Korea; yk.on@samsung.com; 6 Department of Neurology, Samsung Medical Center, Sungkyunkwan University School of Medicine, Seoul 06355, Korea; ohyoung.bang@samsung.com; 7 Pfizer Korea Ltd., Seoul 04631, Korea; ji-min.lee@pfizer.com (J.-M.L.); yoo-jung.park@pfizer.com (Y.-J.P.); ji-soo.shin@pfizer.com (J.-S.S.); seong-sik.kang@pfizer.com (S.K.); 8 Division of Cardiology, Department of Internal Medicine, Korea University, Seoul 02841, Korea

**Keywords:** atrial fibrillation, percutaneous coronary intervention, stents, anticoagulants

## Abstract

We examined antithrombotic treatment patterns with clinical characteristics and therapy changes over time in patients with atrial fibrillation (AF) after percutaneous coronary intervention (PCI). Using the Health Insurance Review and Assessment service claims database (01JAN2007-30NOV2016) in Korea, we included adult patients with AF and PCI: (1) who underwent PCI with stenting between 01JAN2008 and 30NOV2016; (2) with ≥1 claim for AF (ICD code: I48) (3) with antithrombotics 1 day prior to or at the date of PCI; and (4) with CHADS2-VASc of ≥2. In this study, 7749 patients with AF who underwent PCI, triple therapy, dual therapy, dual antiplatelet therapy (DAPT), and single antiplatelet therapy were prescribed to 24.6%, 3.4%, 60.8%, and 11.0%, respectively. In the triple therapy group, 23.1% persisted with triple therapy for 12 months, whereas the remaining patients switched to a different therapy. In the entire cohort and several subgroups, the median treatment duration of triple therapy was 55–87 days. DAPT use for 12 months was the most common treatment pattern (62.6%) in the DAPT group (median treatment duration, 324–345 days). A significant discrepancy exists between the current guidelines and real-world practice regarding antithrombotic treatment with PCI for patients with AF. Appropriate use of anticoagulants should be emphasized.

## 1. Introduction

Atrial fibrillation (AF) is the most common arrhythmia and is complicated by a five-fold increased ischemic stroke risk [1]; therefore, therapeutic prevention with oral anticoagulation (OAC) is required [2,3,4]. Because the prevalence of AF increases with age [2], patients with AF often have several comorbidities. One of the noticeable disorders is coronary artery disease (CAD); it is reported that up to 40% of patients with AF also have CAD, with the majority of them needing percutaneous coronary intervention (PCI) with or without stenting [5]. As they additionally need dual antiplatelet therapy (DAPT), i.e., the combination of aspirin and a P2Y12 inhibitor, to prevent the risk of stent thrombosis, the treatment combined with OAC and DAPT for these patients can be complicated [6].

It has been reported that OAC should be used for preventing thromboembolic events due to AF, whereas DAPT is the best treatment option for preventing stent thrombosis [6,7]. Thus, the regimen combining OAC and DAPT, also known as “triple therapy,” is theoretically the right option for such patients. However, it is expected that this treatment can increase the risk of bleeding; therefore, several randomized controlled trials recently introduced various treatment options, including potential benefits of OAC with single antiplatelet therapy (SAPT), i.e., the so-called dual therapy [8,9,10,11]. Furthermore, some of the most complicated aspects are the duration of each of the treatment combinations; the right timing of changing the combination; the different characteristics of each anticoagulant and antiplatelet; and the patients’ comorbidities, risk factors of bleeding, and procedural risk factors for stent thrombosis. Several guidelines have been rapidly and continuously updated on this topic based on new evidence [4,6,12,13,14]; however, the optimal choice of treatment remains challenging in real-world practice.

Therefore, it is necessary to clearly understand the combination of treatment patterns and longitudinal changes of the treatment in the real world. This study aimed to examine the use of antithrombotic therapy in patients with AF in need of OAC who underwent PCI by evaluating the temporal trends, treatment patterns over time, and treatment duration of each antithrombotic therapy in a nationwide claims database of South Korea.

## 2. Materials and Methods

### 2.1. Data Source

We used data from the Korean Health Insurance Review and Assessment (HIRA) service database, which covers the entire population because of the universal health insurance system in South Korea. Data recorded between January 2007 and November 2016 were used [15]. The data, including patients’ baseline demographics and medical and pharmacy claims, were provided after de-identification [15]. Diagnoses in this database were coded according to the Korean Standard Classification of Diseases, which is based on the International Classification of Diseases 10th Revision (ICD-10) code [15]. The Pusan National University Institutional Review Board determined that this study was exempt from ethical review (PNU IRB/2016_137_HR).

### 2.2. Study Population

**Figure 1:**
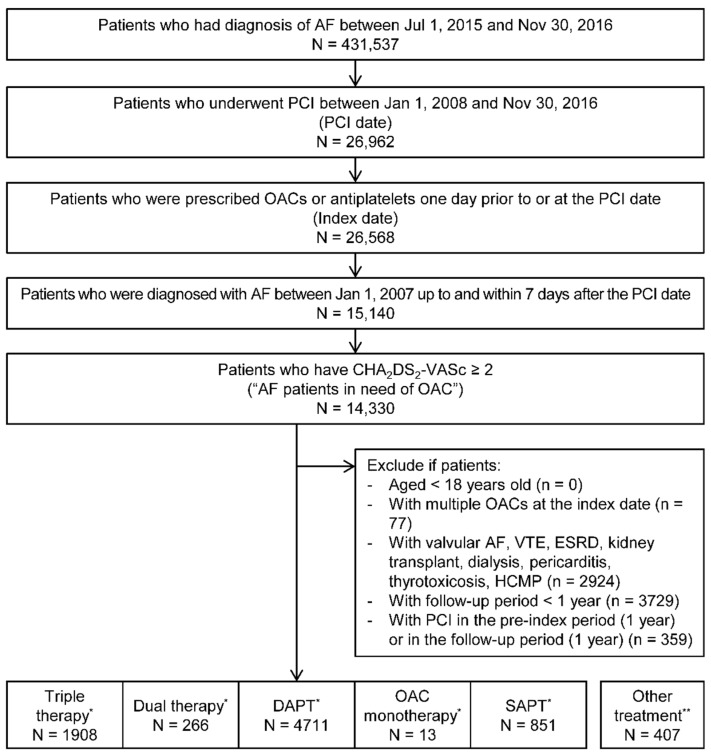
Patient selection flow of patients with atrial fibrillation who underwent percutaneous coronary intervention in need of oral anticoagulants. CHA_2_DS_2_-VASc—congestive heart failure, hypertension, age ≥75 years, diabetes mellitus, stroke, vascular disease, age 65–74 years, and sex. AF, atrial fibrillation; DAPT, dual antiplatelet therapy; ESRD, end-stage renal disease; HCMP, hypertrophic cardiomyopathy; OAC, oral anticoagulants; PCI, percutaneous coronary intervention; SAPT, single antiplatelet therapy; VTE, venous thromboembolism. * Triple therapy means OAC plus DAPT. Dual therapy means OAC plus SAPT. DAPT means aspirin plus clopidogrel/prasugrel/ticagrelor. OAC monotherapy means apixaban, dabigatran, rivaroxaban, or warfarin. SAPT means aspirin, clopidogrel, prasugrel, or ticagrelor. ** Other treatments included the following concomitant uses: clopidogrel plus prasugrel; clopidogrel plus ticagrelor; aspirin plus clopidogrel plus prasugrel; aspirin plus clopidogrel plus ticagrelor; aspirin plus prasugrel plus ticagrelor; and aspirin plus clopidogrel plus prasugrel plus ticagrelor.

We included patients with AF who underwent PCI with stenting and met all of the following criteria: (1) aged ≥18 years at the index date, (2) underwent PCI with stenting between 1 January 2008 and 30 November 2016, (3) had ≥1 claim for AF (ICD code: I48) between 1 January 2007 and 7 days after the date of PCI, (4) were prescribed antithrombotics (either anticoagulant or antiplatelet therapy) 1 day prior to or at the date of PCI, and (5) had CHA_2_DS_2_-VASc ≥ 2 (Figure 1).

The index date was defined as the prescription fill date for antithrombotics with PCI on the same date. If antithrombotics were not prescribed at the same date with PCI but 1 day before the date of PCI, then the prescription fill date for antithrombotics was considered as the index date. Antithrombotics included aspirin, clopidogrel, prasugrel, ticagrelor, apixaban, dabigatran, rivaroxaban, and warfarin. We included patients with AF having CHA_2_DS_2_-VASc of ≥2 who need OAC as per European Society of Cardiology (ESC) guideline.

Patients were excluded if they met one of the following criteria: (1) claims indicating valvular AF, prosthetic heart valves, venous thromboembolism, end-stage chronic kidney disease, kidney transplant, dialysis, pericarditis, thyrotoxicosis, and hypertrophic cardiomyopathy, within 1 year prior to or on the index date; (2) ≥2 OACs at the index date; (3) undergone PCI with stenting within 1 year prior to or after the index date; and (4) a follow-up period of <1 year. Patients were followed up for 1 year.

### 2.3. Temporal Trends and Treatment Patterns

We examined the proportion of antithrombotic treatment and changes in the treatment patterns for 12 months of follow-up period.

We defined antithrombotic treatment by assessing whether antithrombotic therapies fit into one of the following five categories: triple therapy (OAC plus DAPT), dual therapy (OAC plus SAPT), DAPT, OAC monotherapy, and SAPT. Drugs prescribed on the same day were considered as concomitant use (i.e., combination therapy). DAPT was defined as combination therapy with aspirin and clopidogrel (aspirin–clopidogrel), aspirin and prasugrel (aspirin–prasugrel), or aspirin and ticagrelor (aspirin–ticagrelor). SAPT included aspirin, clopidogrel, prasugrel, and ticagrelor. OAC included apixaban, dabigatran, rivaroxaban, and warfarin. Treatment prescribed at the index date was defined as the index treatment. The pattern of antithrombotic treatments was grouped using triple therapy, dual therapy, DAPT, OAC monotherapy, and SAPT as the index treatment, which are referred to as triple therapy group, dual therapy group, DAPT group, OAC monotherapy group, and SAPT group, respectively.

To explore the temporal trends in antithrombotic treatment after PCI, we calculated the proportion of antithrombotic treatment at the index date and specific time point (t) (defined as 30, 60, 90, 120, 150, 180, 210, 240, 270, 300, 330, and 365 days after the index date) as the number of patients in a specific antithrombotic category divided by the total number of the cohort.

We defined the treatment pattern for 12 months as successive combinations of antithrombotic treatment at a series of specific time points for each patient. To define antithrombotic treatment at a specific time point, we assessed whether antithrombotic prescriptions met one of the following criteria: (1) prescription issued in the periods ranged from a previous time point plus 1 day to a specific time point; and (2) the duration of the prescription (as defined by the days of supply plus a 7 day grace period) included one of the specific time points. Because we assumed that the treatment effect lasts for up to 7 days, we allowed a 7 day grace period. Days of supply of the combination therapies (i.e., triple therapy, dual therapy, or DAPT) were calculated based on the minimum days of supply of a drug comprising the combination therapies. For example, if a triple therapy prescription was issued in the period of 1–30 days, or the duration of the triple therapy prescription included the specific time point of 30 days, it was considered that the patients received triple therapy at 30 days after the index date. If two or more categories of antithrombotic therapies were identified at the same specific time point, we employed the following priorities to define only one antithrombotic treatment at a specific time point: triple therapy > dual therapy > DAPT > OAC monotherapy > SAPT (“>” here means “has priority over”).

### 2.4. Treatment Duration

We examined the mean and median treatment duration of index treatment until the discontinuation or switching of therapy occurred. Discontinuation was defined as having no prescriptions of any antithrombotic treatment at the next time point. Switching was defined as changing to another category among five antithrombotic therapies at the next time point. The treatment duration of index treatment was calculated by summing the days of supply of the prescriptions between the index date and a specific time point before discontinuation/switching occurred. For example, if a patient was defined as having triple therapy at 30 days and dual therapy at 60 days, we considered that switching occurred at 60 days. Next, the days of triple therapy prescriptions between the index date and 30 days were summed to calculate the treatment duration of triple therapy. In addition, only consecutive prescriptions were used to calculate the duration of a certain treatment category. For example, if a patient was defined as having triple therapy at 30 days and then switched to dual therapy at 60 days, and was defined as having triple therapy again at 90 days, only prescriptions issued between the index date and 30 days were considered as the treatment duration of triple therapy.

We investigated the treatment duration of the triple therapy and DAPT, i.e., triple therapy/DAPT, as an index treatment immediately after PCI, as per the current guidelines [3,4,6,12,16]. Moreover, we estimated the treatment duration of dual therapy in patients who started with triple therapy and switched to dual therapy, which is the recommended treatment regimen in current guidelines [3].

### 2.5. Statistical Analysis

Descriptive statistics (means, medians, frequencies, and percentages) were calculated.

Baseline characteristics by index treatment were compared using chi-square test for categorical variables or analysis of variance for continuous variables with *p* values. The statistical significance was set at two-tailed *p* < 0.05. The CHA_2_DS_2_-VASc and HAS-BLED score, Charlson Comorbidity Index, and baseline medication use details are provided in Tables S1–S4. Subgroup analyses were conducted in those with acute coronary syndrome (ACS) history, with HAS-BLED of ≥3, and with ischemic stroke or systemic embolism (IS/SE) history. Data were analyzed with SAS version 9.4 (SAS Institute Inc., Cary, NC, USA).

## 3. Results

### 3.1. Baseline Characteristics

The number of patients with AF in need of OAC who underwent PCI with stenting was 8156. Among 8156 patients, 1908 had triple therapy, 266 had dual therapy, 4711 had DAPT, 13 had OAC monotherapy, 851 had SAPT, and 407 had other treatments at the index date (Figure 1). We included 7749 patients for the analysis and excluded the patients that were on other combination treatment therapy (Table S5).

For the triple therapy, dual therapy, DAPT, OAC monotherapy, and SAPT groups, the mean age of patients was 70.7, 69.0, 70.3, 68.9, and 69.7 years; the CHA_2_DS_2_-VASc score was 4.6, 4.1, 4.2, 4.6, and 4.1; the HAS-BLED score was 3.7, 3.4, 3.5, 3.9, and 3.5; and the CCI was 4.1, 3.6, 3.7, 3.8, and 3.5, respectively. The DAPT group had the highest proportion of ACS (70.5%). The OAC monotherapy group had the highest proportion of ischemic stroke (53.8%), followed by the triple therapy group (34.1%). In both the triple therapy group and the DAPT group, aspirin plus clopidogrel was the most common component, followed by aspirin plus ticagrelor (Table 1). The use of aspirin plus ticagrelor has been continuously increasing since 2013. It comprised 5% in the triple therapy group and 13% in the DAPT group in 2015 (Figure S1). Within 1 year before the PCI, 143 (1.8%) patients had a history of NOAC use and 1646 (21.2%) patients had a history of warfarin use. Among patients who had a history of NOAC use, 48.3%, 2.1%, 44.8%, 0.0%, and 4.9% of patients had triple therapy, dual therapy, DAPT, OAC monotherapy, and SAPT as the index treatment, respectively. Among patients who had a history of warfarin use, 57.7%, 8.7%, 26.9%, 0.5%, and 6.2% patients had triple therapy, dual therapy, DAPT, OAC monotherapy, and SAPT as the index treatment, respectively.

### 3.2. Treatment Patterns and Temporal Trends

**Figure 2:**
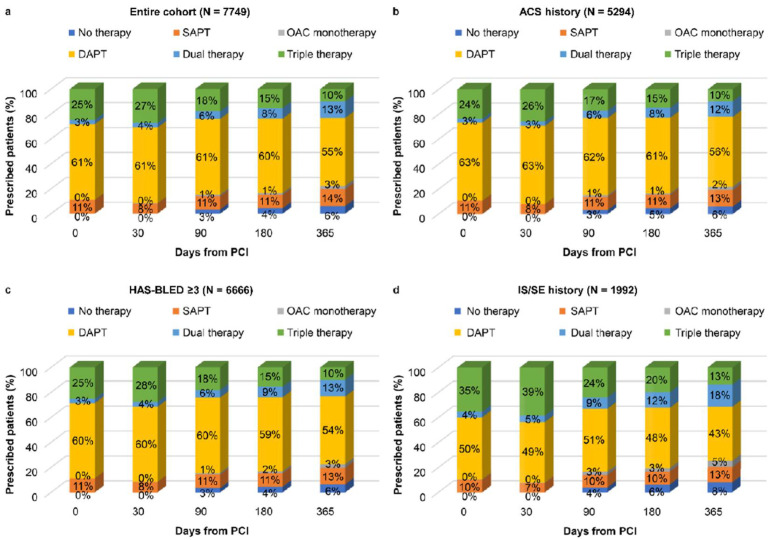
Temporal trends in antithrombotic treatments. (**a**) Entire cohort; (**b**) ACS history; (**c**) HAS-BLED ≥ 3; (**d**) IS/SE history. HAS-BLED—hypertension, abnormal renal and liver function, stroke, bleeding, labile international normalized ratio, elderly, drugs or alcohol. ACS, acute coronary syndrome; DAPT, dual antiplatelet therapy; IS/SE, ischemic stroke/systemic embolism; OAC, oral anticoagulants; PCI, percutaneous coronary intervention; SAPT, single antiplatelet therapy. Triple therapy means OAC plus DAPT. Dual therapy means OAC plus SAPT. DAPT means aspirin plus clopidogrel/prasugrel/ticagrelor. OAC monotherapy means apixaban, dabigatran, rivaroxaban, or warfarin. SAPT means aspirin, clopidogrel, prasugrel, or ticagrelor. Each proportion of antithrombotic therapies in this figure included patients who changed therapies from their index therapies. For example, the proportion of DAPT at 30 days included patients who changed their therapies from other therapies (e.g., SAPT, OAC monotherapy, dual therapy, or triple therapy) to DAPT. Patients’ therapies were cross-sectionally followed-up from the index date to 365 days.

Figure 2 shows temporal trends of antithrombotic therapies after the index date. Each proportion of antithrombotic therapies in Figure 2 included patients who changed therapies from their index therapies, since the patients’ therapies were cross-sectionally followed-up from the index date to 365 days. In the entire cohort, the most common treatment was DAPT (61%), followed by triple therapy (25%), SAPT (11%), dual therapy (3%), and OAC monotherapy (0%) on the index date. During 1 year of follow-up period, the numbers of DAPT and triple therapy users decreased, whereas those of SAPT and dual therapy increased. Compared with the entire cohort, the group with a history of ACS tended to receive DAPT for a relatively long period; the group with a HAS-BLED score of ≥3 tended to receive slightly lower DAPT treatment duration but generally showed a similar trend; the group with a history of IS/SE tended to receive more triple and dual therapy for a relatively longer period (Figure 2).

**Figure 3:**
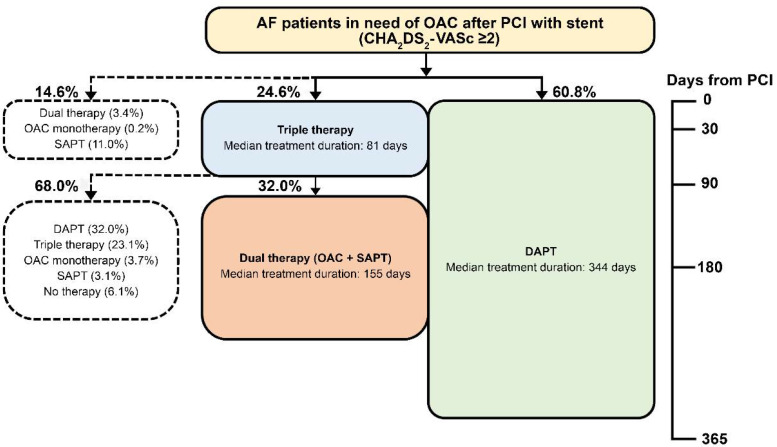
Antithrombotic treatment after percutaneous coronary intervention in patients with atrial fibrillation in need of OAC. CHA_2_DS_2_-VASc—congestive heart failure, hypertension, age ≥ 75 years, diabetes mellitus, stroke, vascular disease, age 65–74 years, and sex. AF, atrial fibrillation; DAPT, dual antiplatelet therapy; OAC, oral anticoagulants; PCI, percutaneous coronary intervention; SAPT, single antiplatelet therapy. Triple therapy means OAC plus DAPT. Dual therapy means OAC plus SAPT. DAPT means aspirin plus clopidogrel/prasugrel/ticagrelor. OAC monotherapy means apixaban, dabigatran, rivaroxaban, or warfarin. SAPT means aspirin, clopidogrel, prasugrel, or ticagrelor. Proportions were calculated from 7749 patients excluding patients with other treatments such as clopidogrel plus prasugrel; clopidogrel plus ticagrelor; aspirin plus clopidogrel plus prasugrel; aspirin plus clopidogrel plus ticagrelor; aspirin plus prasugrel plus ticagrelor; aspirin plus clopidogrel plus prasugrel plus ticagrelor.

In the triple therapy group, 23.1% of patients persisted with triple therapy for 12 months (Table 2), whereas the remaining patients switched (i.e., 32% of patients switched to dual therapy and 32% of patients switched to DAPT; Figure 3). In the DAPT group, DAPT use for 12 months was the most common treatment pattern (62.6%) (Table 2).

### 3.3. Treatment Duration

Compared with the entire cohort, the group with an ACS history had slightly longer treatment duration of triple therapy, dual therapy, and DAPT; the group with the HAS-BLED of ≥3 and IS/SE history had a shorter treatment duration of triple therapy, dual therapy, and DAPT (Table 3).

**Figure 4:**
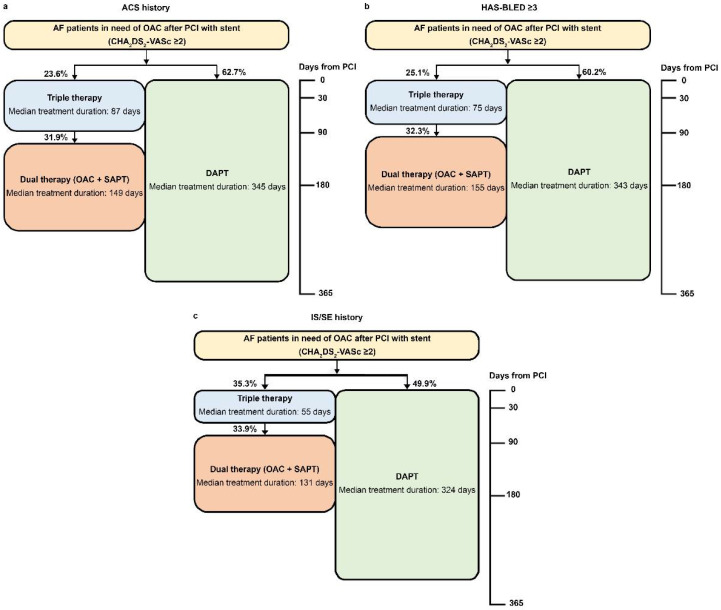
Antithrombotic treatment after percutaneous coronary intervention in patients with atrial fibrillation in need of oral anticoagulants. (**a**) ACS history; (**b**) HAS-BLED ≥ 3; (**c**) IS/SE history. CHA_2_DS_2_-VASc—congestive heart failure, hypertension, age ≥ 75 years, diabetes mellitus, stroke, vascular disease, age 65–74 years, and sex. HAS-BLED—hypertension, abnormal renal and liver function, stroke, bleeding, labile international normalized ratio, elderly, drugs or alcohol. AF, atrial fibrillation; DAPT, dual antiplatelet therapy; IS/SE, ischemic stroke/systemic embolism; OAC, oral anticoagulants; PCI, percutaneous coronary intervention; SAPT, single antiplatelet therapy. Triple therapy means OAC plus DAPT. Dual therapy means OAC plus SAPT. DAPT means aspirin plus clopidogrel/prasugrel/ticagrelor. OAC monotherapy means apixaban, dabigatran, rivaroxaban, or warfarin. SAPT means aspirin, clopidogrel, prasugrel, or ticagrelor. Proportions were calculated from 7749 patients excluding patients who had other treatments.

The median treatment durations of DAPT ranged between 324 and 345 days in the entire cohort (344 days), ACS history group (345 days), HAS-BLED of ≥3 group (343 days), and IS/SE history group (324 days) (Figure 3 and Figure 4). The median treatment durations of triple therapy were 55–87 days in the entire cohort (81 days), ACS history group (87 days), HAS-BLED of ≥3 group (75 days), and IS/SE history group (55 days) (Figure 3 and Figure 4).

## 4. Discussion

This retrospective cohort study showed that there is significant discrepancy between real-world treatment patterns and the current guidelines regarding patients with AF and PCI [4,6,12,13,14]; the majority of patients with AF who needed OAC were prescribed DAPT without OAC immediately after the PCI procedure as an index therapy, and once this choice of treatment was made, the same prescription was largely maintained for 1 year. This trend was even more prevalent in the ACS history group. In addition, even if triple therapy was applied at the initial phase, only 32% of them switched to dual therapy; the remaining patients switched to DAPT, thereby excluding OAC again. In patients who had other treatments, there were some combinations of two or more P2Y12 inhibitors, possibly due to overlapping of prescriptions during the switch from one drug to another. These other treatments were not included in the analyses.

This result may indicate that the importance of using OAC in this AF + PCI group has been somewhat underestimated. Not only during the initial stage of PCI but also at 1 year after the procedure, the priority of antiplatelet therapy was higher than OAC; combining OAC had been a less preferred option for this group. This result may indicate the underlying clinical apprehension in the real world: first, bleeding concerns may affect the tendency to avoid triple therapy prescription, and second, the concerns of ischemic events, particularly related to the stent thrombosis, might be a more important decision factor compared with those associated with AF-related thromboembolism.

It is reported that the incidence of stent thrombosis has been decreasing, owing to the improvement of stent design and procedures [17,18]. Although the reported incidence of stent thrombosis varies depending on clinical settings and definitions, the reported incidences have been decreasing to <1% [19,20]. In addition, there are several procedure-related factors that affect the incidence of stent thrombosis, not just post-PCI antithrombotic treatment. Moreover, the probability of stent thrombosis event decreases dramatically after 30 days from the PCI procedure [21]. Hence, a huge sample size is required to demonstrate statistically significant outcomes of stent thrombosis in a clinical trial setting with various antithrombotic regimens; thus, no definite study has reported regarding this to date.

The risk of stroke due to AF without appropriate anticoagulant is significant; there is a 2.2% risk per year in patients with AF who have a CHA_2_DS_2_-VASc score of 2, and it increases with time [22]. Moreover, the severity of stroke due to AF is greater than that of stroke caused by other pathologies [23]. Therefore, undoubtedly, preventing stroke in patients with AF is crucial even with the PCI procedure experience, and it is already well reported that DAPT is not sufficient for reducing stroke events in this population [7].

Our results indicate that current real-world practice in South Korea might be inadequate to prevent stroke effectively in some patients. Previously, Park et al. also reported about the under-usage of OAC in this patient group [24] after PCI procedure, with ~76.1% of patients who received DAPT, and only 17.1% received OAC, at discharge. Although the time period of the dataset was different, DAPT was the major treatment choice in the first month (61%) of our study; thus, it could be explained that the overall trend of treatment is similar with regard to DAPT as preferred regimen over OAC use.

Fortunately, in addition to previous pivotal RCTs of NOACs, in terms of efficacy (i.e., preventing stroke-related events) outcomes, several recent RCTs in these AF + PCI patients with NOACs cast new light on the bleeding outcomes when it comes to dual therapy [9,10,11,25]. Therefore, this evidence can be considered in treatment options, and overall treatment patterns may change soon. However, clinical decisions for every patient with different clinical characteristics might be still challenging. This research has a few advantages. To the best of our knowledge, there are no real-world studies on exploring the treatment patterns over time at patient level; thus, our study shows the accumulating trend of treatment options in a longitudinal approach, considering individual clinical factors that might affect the decision of prescription and change at each time point. Because there is a previous study on temporal trends of treatment between 2006 and 2015 with annual snapshots [26], we believe this study result provides a complementary point of view. For instance, MI and PCI history were associated with the underuse of triple therapy in the previous report [26], and our study showed that the ACS history group tended to use DAPT for a relatively long period. Based on these results, clinical histories might be one of the important factors for not only the choice of treatment combination in general but also the maintenance period of the treatment in individual cases.

This study has some limitations. First, because claims data were collected for reimbursement purposes, this analysis is subject to the usual limitations inherent to claims data, including coding errors and absence of laboratory data. However, this limitation is of less concern here, because patients who underwent PCI were defined using material codes of coronary stents in this study. Moreover, it had been reported that codes in the HIRA database tended to be more accurate in severe conditions and an inpatient setting [27]. Second, we assumed that patients took all the medications prescribed to them because we could not accurately measure whether patients took their medication or the precise time at which patients discontinued treatment. Third, we could not determine the potential difference in study results between active cancer patients, who may be associated with AF and hypercoagulable state [1], and non-cancer patients. Lastly, a 1 year follow-up period may not be sufficient to observe the comprehensive treatment patterns in this study population; future studies with longer follow-up periods may be helpful in further delineating longer-term outcomes. The cohort used in this study might not represent the current real-world practice pattern as the database used for this analysis represents the treatment pattern during 2015–2016. Since then, the practice has changed substantially following the updated reimbursement guidelines of NOACs.

## 5. Conclusions

There is significant discrepancy between the current guidelines and real-world practice in antithrombotic treatment for patients with AF and PCI. Appropriate use of anticoagulants, including NOACs, should be emphasized.

## Figures and Tables

**Table 1:** Baseline characteristics by index treatment.

	Triple Therapy ^a^ (N = 1908)	Dual Therapy ^a^ (N = 266)	DAPT ^a^ (N = 4711)	OAC Monotherapy ^a^ (N = 13)	SAPT ^a^ (N = 851)	*p*-Value ^b^
Index treatment, %						
Warfarin	94.5	97.7	0	92.3	0	NA
NOAC	5.5	2.3	0	7.7	0	NA
Apixaban	0.8	0.8	0	0	0	NA
Dabigatran	2.3	0.8	0	7.7	0	NA
Rivaroxaban	2.4	0.8	0	0	0	NA
Aspirin–clopidogrel	97.2	0	94.1	0	0	NA
Aspirin–prasugrel	0.5	0	1.0	0	0	NA
Aspirin–ticagrelor	2.4	0	4.9	0	0	NA
Aspirin	0	6.0	0	0	6.3	NA
Clopidogrel	0	92.9	0	0	91.2	NA
Prasugrel	0	0.4	0	0	0.6	NA
Ticagrelor	0	0.8	0	0	1.9	NA
Age (years), mean	70.7	69.0	70.3	68.9	69.7	0.0097
Female, %	33.1	28.2	34.5	30.8	36.0	0.1525
Insurance, %						
NHI	92.7	94.0	92.6	100.0	92.9	0.7763
Medical aid	7.3	6.0	7.4	0.0	7.1	
CHA_2_DS_2_-VASc, mean	4.6	4.1	4.2	4.6	4.1	<0.0001
HAS-BLED, mean	3.7	3.4	3.5	3.9	3.5	<0.0001
CCI, mean	4.1	3.6	3.7	3.8	3.5	<0.0001
Medical history, %						
ACS	65.6	58.3	70.5	38.5	65.9	<0.0001
CHF	44.9	33.1	36.1	46.2	26.8	<0.0001
Hypertension	91.6	92.5	92.2	92.3	92.7	0.8604
Diabetes	64.4	66.2	60.8	53.8	63.8	0.027
Ischemic stroke	34.1	29.3	19.2	53.8	20.6	<0.0001
Systemic embolism	4.4	5.3	2.9	7.7	3.8	0.0125
Vascular disease	42.1	31.6	46.6	38.5	38.9	<0.0001
Renal disease (CKD3/4)	1.5	0.0	0.9	0.0	0.1	0.0053
Bleeding	27.1	16.5	26.3	38.5	23.3	0.0012
Medication history, %						
NOACs	3.6	1.1	1.4	0.0	0.8	<0.0001
Warfarin	49.8	53.8	9.4	69.2	12.0	<0.0001
NSAIDs	81.4	80.1	83.3	76.9	83.4	0.2617
Antiplatelets	63.9	64.3	72.5	84.6	70.9	<0.0001
Antiarrhythmics	61.4	56.8	59.4	76.9	61.6	0.1971
Statins	88.7	78.2	88.4	92.3	82.0	<0.0001
Proton pump inhibitors	44.3	30.1	42.6	38.5	28.4	<0.0001
H2-receptor antagonists	74.6	69.2	76.7	53.8	76.0	0.0095
Digoxin	39.8	38.7	29.8	38.5	29.6	<0.0001

CHA_2_DS_2_-VASc—congestive heart failure, hypertension, age ≥ 75 years, diabetes mellitus, stroke, vascular disease, age 65–74 years, and sex. HAS-BLED—hypertension, abnormal renal and liver function, stroke, bleeding, labile international normalized ratio, elderly, drugs or alcohol. ACS, acute coronary syndrome; CCI, Charlson Comorbidity Index; CHF, congestive heart failure; DAPT, dual antiplatelet therapy; CKD, chronic kidney disease; NOAC, non-vitamin K antagonist oral anticoagulants; NSAIDs; nonsteroidal anti-inflammatory drugs; OAC, oral anticoagulants; SAPT, single antiplatelet therapy. ^a^ Triple therapy means OAC plus DAPT. Dual therapy means OAC plus SAPT. DAPT means aspirin plus clopidogrel/prasugrel/ticagrelor. OAC monotherapy means apixaban, dabigatran, rivaroxaban, or warfarin. SAPT means aspirin, clopidogrel, prasugrel, or ticagrelor. ^b^ Chi-square test for categorical variables or analysis of variance for continuous variables.

**Table 2:** Top three treatment patterns in patients with triple therapy or DAPT.

	N	%	Treatment Patterns
Triple therapy group	1908		
1st	440	23.1	Triple therapy 12 months
2nd	218	11.4	Triple therapy 1 month ⇒ DAPT 11 months
3rd	86	4.5	Triple therapy 1 month ⇒ Dual therapy 11 months
Triple therapy group ⇒ Dual therapy switcher	611		
1st	86	14.1	Triple therapy 1 month ⇒ Dual therapy 11 months
2nd	44	7.2	Triple therapy 8 months ⇒ Dual therapy 4 months
3rd	41	6.7	Triple therapy 7 months ⇒ Dual therapy 5 months
DAPT group	4711		
1st	2951	62.6	DAPT 12 months
2nd	119	2.5	DAPT 1 month ⇒ SAPT 11 months
3rd	55	1.2	DAPT 11 months ⇒ SAPT 1 month

DAPT, dual antiplatelet therapy.

**Table 3:** Treatment duration (days) in patients with triple therapy or dual antiplatelet therapy.

			Treatment Durations (Days)	
	N	%	Mean (SE)	Median
Triple therapy group	1908		Treatment duration of triple therapy ^a^	
Entire ^b^	1907	99.9	138.18 (3.17)	81
ACS history ^b^	1251	65.6	141.39 (3.94)	87
HAS-BLED ≥ 3 ^b^	1675	87.8	134.56 (3.37)	75
IS/SE history	703	36.8	122.93 (5.09)	55
Female	694	36.4	120.71 (5.09)	47
Age ≥ 75	632	33.1	122.32 (5.3)	56.5
Triple therapy group ⇒ Dual therapy switcher	611		Treatment duration of dual therapy ^a^	
Entire	611	100.0	162.57 (4.17)	155
ACS history	400	65.5	164.17 (5.21)	149
HAS-BLED ≥ 3	541	88.5	162.53 (4.47)	155
IS/SE history	238	39.0	157.52 (6.99)	131
Female	230	37.6	160.49 (6.81)	150.5
Age ≥ 75	205	33.6	157.49 (7.09)	142
DAPT group	4711		Treatment duration of DAPT ^a^	
Entire	4711	100.0	261.75 (1.99)	344
ACS history	3321	70.5	262.37 (2.38)	345
HAS-BLED ≥ 3	4015	85.2	258.21 (2.19)	343
IS/SE history	994	21.1	236.71 (4.63)	324
Female	1677	35.6	245.93 (3.49)	334
Age ≥ 75	1627	34.5	256.58 (3.44)	341

HAS-BLED—hypertension, abnormal renal and liver function, stroke, bleeding, labile international normalized ratio, elderly, drugs or alcohol. ACS, acute coronary syndrome; DAPT, dual antiplatelet therapy; IS/SE, ischemic stroke/systemic embolism. ^a^ Only consecutive treatments were used to calculate treatment durations. ^b^ There was one missing value in treatment duration of triple therapy.

## Data Availability

The study data were extracted and analyzed from the Korea Health Insurance Review and Assessment Service (HIRA) claims database, and additional data may be obtained from third party (with appropriate authorization approval) but are not publicly available.

## Supplementary Materials

The following are available online at https://www.mdpi.com/article/10.3390/healthcare9091185/s1, Additional file 1: Table S1. Scoring and International Classification Disease, 10th Revision (ICD-10) codes for the factors included in the CHA_2_DS_2_-VASc score, Additional file 2: Table S2. Scoring and *International Classification Disease, 10th Revision* (ICD-10) codes for the factors included in the HAS-BLED score, Additional file 3: Table S3. Scoring and *International Classification Disease, 10th Revision* (ICD-10) codes for the factors included in the Charlson Comorbidity Index, Additional file 4: Table S4. Types of baseline medications, Additional file 5: Table S5. Other treatments, Additional file 6: Figure S1. Proportion of triple therapy user and dual antiplatelet therapy user by year. (a) Triple therapy user by year; (b) DAPT user by year.
