# Supplementary material for: Treatment Pattern of Antithrombotic Therapy over Time after Percutaneous Coronary Intervention in Patients with Atrial Fibrillation in Real-World Practice in Korea
Source: Healthcare (Basel). 2021 Sep 9;9(9):1185. doi: 10.3390/healthcare9091185 (PMC8472294; doi:10.3390/healthcare9091185)
Supplement: Supplementary file 1 [file healthcare-09-01185-s001.zip › healthcare-1320656-supplementary.pdf]

healthcare

Table S1. Scoring and *International Classification of Disease, Tenth Revision* (ICD-10) codes for the factors included in the CHA_2DS_2-VASc score

Condition	ICD-10 codes	Point
Congestive heart failure	I50	1
Hypertension	I10-I15	1
Age	≥75 years	2
Diabetes	E10–E14	1
Stroke	I63, I693 and G459	2
Vascular disease	I220, I221, I228, I229, I742, I743, I744, I21, I252, I700, I7000, I7001, I7380, I7388, and I739	1
Age	65–74 years	1
Sex	Female	1

Table S2. Scoring and *International Classification of Disease, Tenth Revision* (ICD-10) codes for the factors include in the HAS-BLED score

Condition	ICD-10 codes	Point
Hypertension	I10–I15	1
Abnormal renal disease	N183 and N184	1
Abnormal liver function	B15–B19, C22, D684, I982, I983, K70–K77 and Z944	1
Stroke	I63, I693 and G459	1
Bleeding history or predisposition*	I60, I61, I62, I690, I691, I692, S064, S065, S066, S068, I850, I983, K2211, K226, K228, K250, K252, K254, K256, K260, K262, K264, K266, K270, K272, K274, K276, K280, K282, K284, K286, K290, K3181, K5521, K625, K920, K921, K922, D62, H448, H356, H313, H210, H113, H052, H470, H431, I312, N020-N029, N421, N831, N857, N920, N923, N930, N938, N939, M250, R233, R040, R041, R042, R048, R049, T792, T810, N950, R31, R58, T455, Y442, D683, D500, D508, D509, D510, D511, D512, D513, D518, D519, D520, D521, D528, D529, D530, D531, D532, D538, D539, D550, D551, D552, D553, D558, D559, D580, D581, D582, D588, D589, D590, D591,	1

**Condition**	**ICD-10 codes**	**Point**
	D592, D593, D594, D595, D596, D598, D599, D600, D601, D608, D609, D611, D612, D613, D619, D62, D630, D638, D640, D641, D642, D643, D644, and D649	
Elderly	≥65 years	1
Drug therapy	Antiplatelets and NSAIDs	1
Alcoholism	E244, F10, G312, G621, G721, I426, K292, K70, K860, O354, P043, Q860, T510, X45, X65, Y15, Y90-Y91, Z502, Z714 and Z721	1

* A blood transfusion was also required to define a history of bleeding from other sites

Table S3. Scoring and *International Classification of Disease, Tenth Revision* (ICD-10) codes for the factors included in the Charlson Comorbidity Index

Condition	ICD-10 codes	Point
Cerebrovascular disease	G45.x, G46.x, H34.0 and I60.x–I69.x	1
Congestive heart failure	I09.9, I11.0, I13.0, I13.2, I25.5, I42.0, I42.5-I42.9, I43.x, I50.x and P29.0	1
Chronic pulmonary disease	I27.8, I27.9, J40.x–J47.x, J60.x–J67.x, J68.4, J70.1 and J70.3	1
Dementia	F00.x–F03.x, F05.1, G30.x and G31.1	1
Diabetes without chronic complication	E10.0, E10.1, E10.6, E10.8, E10.9, E11.0, E11.1, E11.6, E11.8, E11.9, E12.0, E12.1, E12.6, E12.8, E12.9, E13.0, E13.1, E13.6, E13.8, E13.9, E14.0, E14.1, E14.6, E14.8 and E14.9	1
Mild liver disease	B18.x, K70.0-K70.3, K70.9, K71.3-K71.5, K71.7, K73.x, K74.x, K76.0, K76.2-K76.4, K76.8, K76.9 and Z94.4	1
Myocardial infarction	I21.x, I22.x and I25.2	1
Peripheral vascular disease	I70.x, I71.x, I73.1, I73.8, I73.9, I77.1, I79.0, I79.2, K55.1, K55.8, K55.9, Z95.8 and Z95.9	1
Peptic ulcer disease	K25.x–K28.x,	1
Rheumatologic disease	M05.x, M06.x, M32.x–M34.x M31.5M35.1, M35.3 and M36.0	1
Diabetes with chronic complication	E10.2–E10.5, E10.7, E11.2–E11.5, E11.7, E12.2-E12.5, E12.7, E13.2–E13.5, E13.7, E14.2–E14.5 and E14.7	2
Hemiplegia or paraplegia	G04.1, G11.4, G80.1, G80.2, G81.x, G82.x, G83.0-G83.4 and G83.9	2
Any malignancy including leukaemia and lymphoma	C00.x–C26.x, C30.x–C34.x, C37.x–C41.x, C43.x, C45.x–C58.x, C60.x–C76.x, C81.x–C85.x, C88.x and C90.x–C97.x	2
Renal disease	I12.0, I13.1, N03.2-N03.7, N05.2-N05.7, N18.x, N19.x, N25.0, Z49.0-Z49.2, Z94.0 and Z99.2	2
Moderate or severe liver disease	I85.0, I85.9, I86.4, I98.2, K70.4, K71.1, K72.1, K72.9, K76.5, K76.6 and K76.7	3
AIDS/HIV	B20.x–B22.x and B24.x	6
Metastatic solid tumour	C77.x–C80.x	6

Table S4. Types of baseline medications

Class	Drugs
NSAIDs	Bromfenac, celecoxib, diclofenac, etodolac, fenoprofen, flurbiprofen, ibuprofen, indomethacin, ketoprofen, ketorolac, naproxen, meclofenamate, mefenamic acid, meloxicam, nabumetone, oxaprozin, piroxicam, sulindac and tolmetin
Antiplatelets	Aspirin, clopidogrel, prasugrel, ticlopidine, cilostazol, abciximab, tirofiban, dipyridamole and ticagrelor
Proton pump inhibitors	Omeprazole, pantoprazole, lansoprazole, rabeprazole, esomeprazole and dexlansoprazole
H_2-receptor antagonists	Cimetidine, ranitidine, famotidine, nizatidine, roxatidine and lafutidine
Antiarrhythmics	Quinidine, procainamide, mexiletine, propafenone, flecainide, amiodarone, bretylium, dronedarone, propranolol, atenolol, esmolol, verapamil, diltiazem and sotalol
Digoxin	Digoxin
Statins	Atorvastatin, fluvastatin, lovastatin, pitavastatin, pravastatin, rosuvastatin and simvastatin

NSAID, nonsteroidal anti-inflammatory drug

Table S5. Other treatments

	Other treatments				
	OAC-TAPT (N = 144)	QAPT (N = 1)	TAPT (N = 276)	OAC + Dual $P2Y_{12}$ inhibitors (N = 5)	Dual $P2Y_{12}$ inhibitors (N = 11)
Index treatment, %					
Warfarin	81.6	0.0	0.0	60.0	0.0
NOAC	18.4	0.0	0.0	40.0	0.0
Apixaban	3.5	0.0	0.0	0.0	0.0
Dabigatran	9.6	0.0	0.0	0.0	0.0
Rivaroxaban	5.3	0.0	0.0	40.0	0.0
Aspirin-Clopidogrel	0.0	0.0	0.0	0.0	0.0
Aspirin-Prasugrel	0.0	0.0	0.0	0.0	0.0
Aspirin-Ticagrelor	0.0	0.0	0.0	0.0	0.0
Clopidogrel-Prasugrel	0.0	0.0	0.0	20.0	45.5
Clopidogrel-Ticagrelor	0.0	0.0	0.0	80.0	54.5
Aspirin-Clopidogrel-Prasugrel	12.3	0.0	16.7	0.0	0.0
Aspirin-Clopidogrel-Ticagrelor	86.8	0.0	81.5	0.0	0.0
Aspirin-Prasugrel-Ticagrelor	0.9	0.0	1.8	0.0	0.0
Aspirin-Clopidogrel-Prasugrel-Ticagrelor	0.0	100.0	0.0	0.0	0.0

TAPT, triple antiplatelet therapy; NOAC, nonvitamin K antagonist oral anticoagulants; OAC, oral anticoagulants; QAPT, quadruple antiplatelet therapy

Figure S1. Proportion of triple therapy user and dual antiplatelet therapy user by year.

(a) Triple therapy user by year; (b) DAPT user by year.

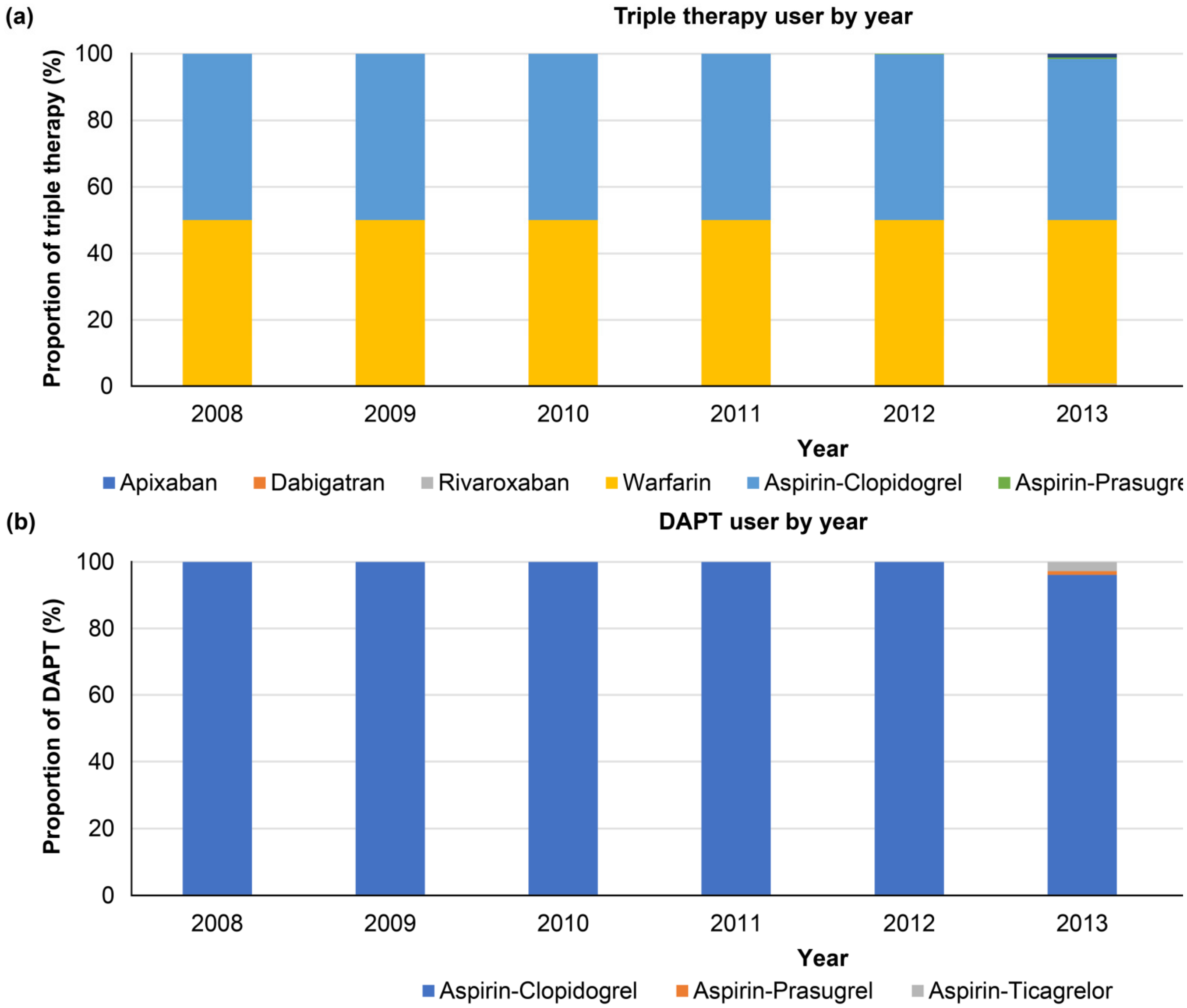

DAPT, dual antiplatelet therapy

*Triple therapy means OAC plus DAPT. DAPT means aspirin plus clopidogrel/prasugrel/ticagrelor.
